# A cutoff thyroglobulin value suggestive of distant metastases in differentiated thyroid cancer patients

**DOI:** 10.1590/1414-431X20209781

**Published:** 2020-10-09

**Authors:** J.S. Couto, M.F.O. Almeida, V.C.G. Trindade, M.M.S. Marone, N.M. Scalissi, A.N. Cury, C. Ferraz, R.P. Padovani

**Affiliations:** 1 Serviço de Endocrinologia e Metabologia, Departamento de Medicina, Irmandade da Santa Casa de Misericórdia de São Paulo, São Paulo, SP, Brasil; 2 Serviço de Medicina Nuclear, Nuclimagem, São Paulo, SP, Brasil; 3 Faculdade de Ciências Médicas da Santa Casa de São Paulo, São Paulo, SP, Brasil

**Keywords:** Thyroid cancer, Stimulated thyroglobulin, Cutoff point, High risk, Distant metastasis

## Abstract

Serum thyroglobulin is used as part of the early postoperative assessment of differentiated thyroid cancer (DTC) since there is a clear relationship between an increased risk of recurrence and persistent disease after initial treatment and high postoperative stimulated thyroglobulin (ps-Tg) values. Thus, although ps-Tg above 10–30 ng/mL is considered an independent predictor of worse prognosis, the value that is associated with distant metastases is not defined. Thus, this was our objective. We selected 655 DTC patients from a nuclear medicine department database (Irmandade Santa Casa de Misericórdia de São Paulo, Brazil). All patients had received total thyroidectomy and radioactive iodine (RAI) therapy and had ps-Tg values higher than 10 ng/mL with negative anti-thyroglobulin antibodies. Then, we selected patients who presented post-therapy whole-body scan with pulmonary and/or bone uptake but with no mediastinum or cervical uptake. Patients with negative findings on functional imaging or any doubt on lung/bone uptake were submitted to additional exams to exclude another non-thyroid tumor. Of the 655 patients, 14.3% had pulmonary and 4.4% bone metastases. There was a significant difference in ps-Tg levels between patients with and without metastases (P<0.001). The cutoff value of ps-Tg was 117.5 ng/mL (sensitivity: 70.2%; specificity: 71.7%) for those with lung metastasis, and 150.5 ng/mL (sensitivity: 79.3%; specificity: 85%) for those with bone metastasis. The cutoff value for patients with eitherpulmonary or bone metastasis was 117.5 ng/mL (sensitivity: 70.2%; specificity: 83.7%). Our findings demonstrated that ps-Tg could predict distant metastasis in DTC patients. We identified a cutoff of 117.5 ng/mL with a high negative predictive value of 93.7%.

## Introduction

Thyroid cancer is a rare neoplasia, accounting for about 1% of all cancers. However, it is the most common tumor among malignant endocrine neoplasms (1,2). Differentiated thyroid carcinoma (DTC), including papillary, follicular, and Hürthle cell thyroid carcinoma, accounts for more than 90% of all thyroid neoplasms (3,4). Statistical data show an increase in the incidence of thyroid cancer in recent decades (1,2). Over the past 10 years, the incidence rate of thyroid cancer has increased by an average of 3.1% each year in the US (3). Nevertheless, the mortality rate is relatively stable (1–4), increasing, on average, only 0.7% per year between 2006 and 2015. The 5-year survival rate for thyroid cancer is 98.1% (3). Although the overall response to treatment is frequently excellent, the rate of recurrent or persistent cases varies from 23 to 30% (5).

Adequate post-operative risk assessment is crucial for follow-up and therapeutic planning strategies to distinguish patients who may require more aggressive therapies compared to those who have an indolent course, minimizing treatment-related morbidity. Therefore, long-term follow-up is necessary for all patients, especially those at high risk of metastasis and recurrent disease.

Patients with DTC who develop distant metastases usually have a worse prognosis (6,7). The most common sites of distant metastasis are the lungs and bones (4,8,9), and the early diagnosis of these metastases is essential for adequate treatment and a better response to therapy (8).

Over the years, several staging systems have been developed to guide initial management of DTC. While the American Joint Committee on Cancer (AJCC)/TNM staging system provides information with regard to disease-specific mortality, the American Thyroid Association (ATA) risk stratification system uses clinicopathological features to classify DTC patients as having either low, intermediate, or high risk of either recurrence or persistent disease (4,10).

However, in addition to the staging system, the status of the disease in the postoperative period is also of relevant importance in deciding the treatment to be proposed. As part of this assessment, the postoperative stimulated thyroglobulin (ps-Tg) measurement is considered relevant.

Several studies have demonstrated the relationship between increased risk of recurrence and persistent disease after total thyroidectomy and ablation of the remaining radioactive iodine (RAI) and high ps-Tg values (11–15). In addition, ps-Tg is often considered an independent predictor of persistent or recurrent disease (13–15).

However, although high ps-Tg values in the postoperative period (>10–30 ng/mL) may be associated with worse survival (11,12), and ps-Tg values greater than 5-10 ng/mL correlate with the increased probability of avid ^131^I metastatic disease in the postoperative period (16–18), the ps-Tg value associated with distant metastases at the time of the postoperative evaluation is not defined.

To date, few studies (19–24) have evaluated the predictive value of ps-Tg associated with distant metastasis, excluding those patients with only cervical metastatic disease. Since detecting distant, metastasizing cancer cells in the clinical setting is of high relevance for treatment decision, any additional knowledge on refining the prognostic indicators of a patient’s postoperative risk such as ps-Tg will be a valuable tool for improving patient outcome.

This study aimed to determine the ps-Tg value that identifies distant metastasis and, thereby, high-risk patients more accurately.

## Material and Methods

**Figure 1:**
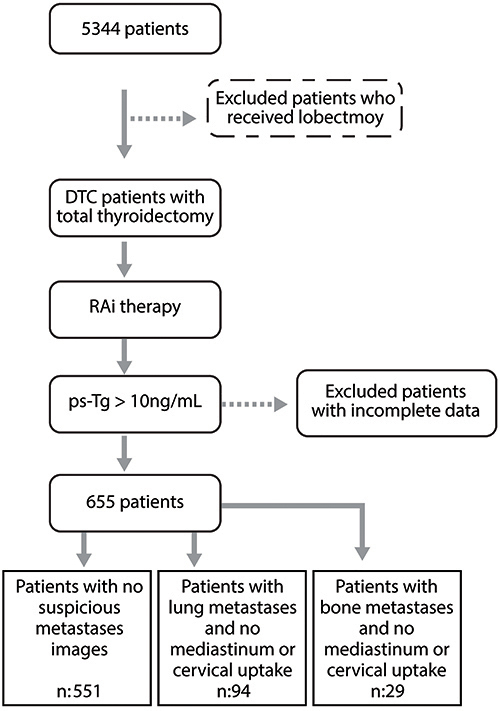
Flow chart of inclusion-exclusion of patients in the present study. DTC: differentiated thyroid cancer; RAI: radioactive iodine; ps-Tg: postoperative stimulated thyroglobulin.

We selected 655 DTC patients from a total of 5344 patients (Figure 1) referred to the Nuclear Medicine Department at Santa Casa de São Paulo from 1972-2015. As we were looking for high-risk patients with distant metastases, we initially selected just patients who had undergone total thyroidectomy and RAI therapy and also had ps-Tg values higher than 10 ng/mL with negative anti-thyroglobulin antibodies. Since previous studies have already shown an association between ps-Tg values (>10–30 ng/mL) and poor overall survival, lower disease-free survival (11,13,25,26), and, in some multivariate analyses, higher risk of persistent or recurrent disease (13,15,16), we used this parameter to initiate our patient selection.

After the exclusion of all patients with incomplete data, we selected patients who presented post-therapy whole-body scan with pulmonary and/or bone uptake but with no mediastinum or cervical uptake. As this research was carried out in a nuclear medicine service and based on information collected from a database, not all patients had an image exam confirming bone or lung metastasis. However, patients with negative findings on functional imaging or any doubt on lung/bone uptake were submitted to additional exams to exclude another non-thyroid tumor. Thus, our analysis group consisted of 655 patients.

This was a retrospective analysis. Patients’ information was obtained from a database of a nuclear medicine service to which patients from various health centers are directed for treatment and exams. As many did not return for follow-up or subsequent exams, we could not include many of them.

The study was approved by the research ethics committee of Santa Casa de São Paulo. The requirement for informed consent was waived, since there were no interventions on patients and most individuals no longer visited the institution.

In order to improve the quality of the description and presentation of the study results, we followed the STROBE Statement (Strengthening the Reporting of Observational Studies in Epidemiology) (27).

Levels of ps-Tg were measured at least four weeks after surgery, before radioimmunotherapy (RIT), and after withdrawal of levothyroxine. The patients were in a state of relative hypothyroidism with thyroid stimulating hormone (TSH) levels ≥30 μIU/mL, determined using chemiluminescence immunoassay. Serum Tg levels were determined using the Immulite Tg assay (Roche Diagnostics, Germany). This is a sensitive two-site chemiluminescent immunoassay and the lower limit of detection was 0.2 ng/mL. Anti-Tg levels were determined using electrochemiluminescence immunoassay (Roche Diagnostics GmbH). All samples were analyzed in the same laboratory.

Patient characteristics, including age and gender, in addition to pathological characteristics, such as histology subtypes, tumor size, presence of multifocality, vascular, lymphatic, neural, or thyroid capsule invasion, presence of either minimal and gross extra thyroidal extension or lymph node metastasis, surgery type, TSH, ps-Tg levels, and the formation of bone and pulmonary metastases were compiled.

The presence of any of the following was considered as distant metastasis: 1) distant metastatic lesions confirmed by pathology; 2) negative findings on functional imaging, but structural lesions suggested by tomography, magnetic resonance imaging, or bone scintigraphy, or 18F-FDG PET/CT; and 3) focal or diffuse uptake in distant metastatic lesions on whole body scans after excluding contamination and physiological iodine uptake, with or without positive findings in other complementary imaging modalities.

Data collection was carried out through database analysis. Mann-Whitney test or Student's *t*-test were used to compare the groups. Comparison of ps-Tg by neck dissection and by type of surgery was carried out using median tests. The normality in the distribution of variables was verified using the Kolmogorov-Smirnov test.

The cutoff values of ps-Tg were obtained using receiver operating characteristic (ROC) analysis. All statistical analyses were carried out with SPSS software (Statistical Package for Social Sciences, Version 20.0, IBM, USA), and statistical significance was set at a P-value <0.05.

## Results

The descriptive characteristics of the 655 patients enrolled in this study are reported in Table 1. Most of the study population was female (79.7%) and younger than 45 years at diagnosis. Histological examination indicated that a papillary pattern (92.1%) with the classic variant was the most common subtype. Regarding initial therapy, 73.4% of patients had total thyroidectomy without neck dissection. Microcarcinomas represented 25.3% of tumors and a minority of tumors were larger than 4 cm (17.7%). The mean radioiodine dose used was 221.6 mCi and the median time between surgery and the administration of the first dose was 7 months.

**Table 1:** Clinical and histopathologic profile of 655 patients with differentiated thyroid cancer.

Features	Number of patients	Percent
Gender		
Female	522	79.69
Age (years)	Mean: 44.2 ± 15.1	
Histological type		
Papillary carcinoma	603	92.06
Follicular carcinoma	34	5.19
Oncocytic Carcinoma	18	2.7
Papillary variant		
Classical	387	64.18
Follicular	192	31.84
Others	24	3.98
Follicular subclassification		
Minimally invasive	27	79.41
Extensively invasive	7	20.59
Tumor size	Mean: 2.6 ± 2.1 cm	
≤1 cm	166	25.34
>1 and ≤4 cm	373	56.95
>4 cm	116	17.71
Multifocality	186	28.39
Tumor capsule invasion	83	12.67
Thyroid capsule invasion	142	21.68
Lymphatic invasion	150	22.90
Vascular invasion	181	27.63
Neural invasion	29	4.43
Minimal extra thyroidal extension	167	25.49
Gross extra thyroidal extension	40	6.10
Lymph node metastasis	246	37.55
Type of surgery		
Total thyroidectomy	481	73.44
Total thyroidectomy + central neck dissection	63	9.62
Total thyroidectomy + ipsilateral neck dissection	35	5.34
Total thyroidectomy + bilateral neck dissection	76	11.60
Iodine dose	Mean: 221.6 ± 59.9 mCi	
Time from surgery to radioiodine therapy	Median: 7 months	
Uptake of iodine in whole body scans	Mean: 1.7 ± 4.6%	
Metastases	104	15.88
Pulmonary	94	14.35
Bone	29	4.43

Overall, 104 (15.88%) patients had distant metastases, of which 94 (14.3%) presented with lung metastasis and 29 (4.4%) with bone metastasis; only 19 patients (2.9%) had both metastases (Table 1).

According to Supplementary Table S1, an association was observed between distant metastasis and ps-Tg levels (P<0.001). Thus, a greater percentage of distant metastasis was noted in patients with ps-Tg above 100 ng/mL compared to those with ps-Tg between 10 and 30 ng/mL (41.6 *vs* 2.8%). Corroborating this result, the mean ps-Tg in the group with distant metastases was higher than the group without this condition. Groups with and without distant metastases were compared regarding the main clinical and pathological aspects (Supplementary Table S1).

To show that ps-Tg added diagnostic value to DTC patients, we ran a multivariate analysis to investigate if this was an independent prognostic factor. Using logistic regression for the outcome of lung and/or bone metastasis, the variables gender, age, histological type, and tumor size were evaluated. Other pathological characteristics of higher recurrence risk such as presence of multifocality, vascular, lymphatic, and neural invasion, thyroid capsule invasion, or presence of either minimal and gross extra thyroidal extension or lymph node metastasis, and ps-Tg were also included in the analysis. The only independent prognostic factor found was ps-Tg (P<0.001) (Table 2), excluding other possible independent risk factors for metastasis.

**Table 2:** Prognostic factors for distant metastases in patients with differentiated thyroid cancer.

	Univariate analysis		Multivariate analysis	
	OR (95%CI)	P	OR (95%CI)	P
ps-Tg range (reference 10-30) (ng/mL)		<0.001		<0.001
30.1–50	3.36 (1.21–9.33)	0.020	3.60 (1.27–10.22)	0.016
50.1–100	6.92 (2.88–16.64)	<0.001	7.24 (2.95–17.78)	<0.001
>100	24.27 (11.73–50.20)	<0.001	26.02 (12.3–55.02)	<0.001
ps-Tg (ng/mL)	1.0005 (1.0003–1.0008)	<0.001	–	–
Male	1.06 (0.64–1.78)	0.815	0.90 (0.50–1.64)	0.741
Age (years)	1.01 (1.00–1.02)	0.203	1.00 (0.98–1.01)	0.881
Size ranges (reference ≥4cm)		0.689		0.609
≤1 cm	1.11 (0.56–2.18)	0.766	1.26 (0,58–2.76)	0.563
1–4 cm	1.27 (0.70–2.30)	0.429	1.41 (0.71–2.81)	0.324
Size (cm)	0.98 (0.89–1.08)	0.710	–	–
Multifocality	1.08 (0.69–1.72)	0.728	1.07 (0.61–1.85)	0.820
Tumor capsule invasion	1.09 (0.59–2.01)	0.792	1.32 (0.63–2.74)	0.461
Thyroid capsule invasion	1.03 (0.62–1.71)	0.906	1.13 (0.62–2.08)	0.683
Lymphatic invasion	1.22 (0.75–1.98)	0.419	1.29 (0.65–2.54)	0.470
Vascular invasion	1.27 (0.80–1.99)	0.309	1.06 (0.56–2.01)	0.864
Neural invasion	0.84 (0.29–2.47)	0.754	0.52 (0.14–1.89)	0.318
Minimal extra thyroidal extension	0.91 (0.56–1.49)	0.710	0.56 (0.30–1.06)	0.075
Gross extra thyroidal extension	1.85 (0.87–3.91)	0.108	1.72 (0.68–4.38)	0.252
Lymph node metastasis	1.27 (0.83–1.94)	0.276	1.51 (0.88–2.58)	0.134
Number of lymph node metastasis	1.01 (0.98–1.04)	0.530	–	–
Type of surgery (reference Total thyroidectomy)		0.120		–
Total thyroidectomy	1.61 (0.83–3.12)	0.161	–	–
Total thyroidectomy + central neck dissection	1.28 (0.51–3.20)	0.599	–	–
Total thyroidectomy + ipsilateral neck dissection	1.92 (1.06–3.45)	0.030	–	–
Neck dissection	1.67 (1.07–2.61)	0.024	1.38 (0.81–2.37)	0.237

OR: odds ratio; ps-Tg: postoperative stimulated thyroglobulin. Hosmer and Lemeshow test for the final multivariate analysis (P=1.000; n=655).

Regarding the type of initial therapy, when we compared the median ps-Tg values between patients who underwent total thyroidectomy and any type of neck dissection, we did not observe a statistically significant difference between the medians of ps-Tg values (P=0.07), confirming the clear relationship between distant metastases and the ps-Tg values found in our analysis (Table 3).

**Table 3:** Summary of postoperative stimulated thyroglobulin (ps-Tg) (ng/mL) levels by type of surgery and neck dissection in patients with differentiated thyroid cancer.

	Mean	Median	n	P
Type of surgery				0.077
Total thyroidectomy	504.55	28.56	481	
Total thyroidectomy + central neck dissection	298.78	47.92	63	
Total thyroidectomy + ipsilateral neck dissection	163.15	31.80	35	
Total thyroidectomy + bilateral neck dissection	457.40	47.17	76	

Non-parametric test was used to compare medians.

**Figure 2:**
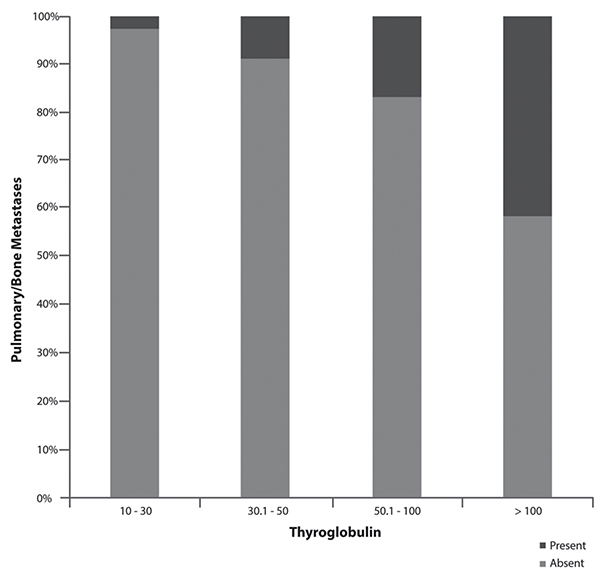
Distribution of distant pulmonary and bone metastases according to thyroglobulin levels. P<0.05 between present and absent at all thyroglobulin levels (chi-squared test).

**Figure 3:**
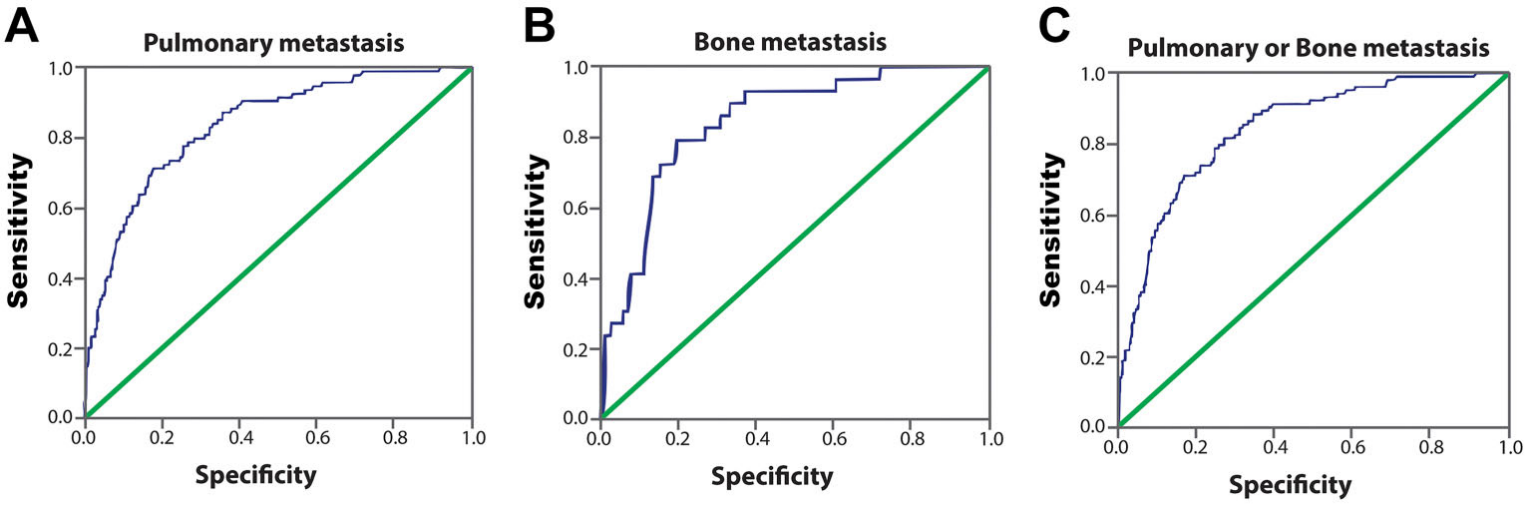
ROC of serum postoperative stimulated thyroglobulin (ps-Tg). ROC in identifying the presence of pulmonary metastasis (**A**), bone metastasis (**B**), and either pulmonary or bone metastasis (**C**). The area under the curve (AUC) for the ps-Tg level used for identifying metastasis was 0.836 (pulmonary), 0.847 (bone), and 0.845 (pulmonary or bone).

Looking to determine a ps-Tg value that identified distant metastasis and, thereby, high-risk patients more accurately, we divided the patients into groups regarding ps-Tg using the values 10–30, 30.1–50, 50.1–100, and >100 ng/mL (11–13,19,22,28). When we compared the groups, we observed that in the range of 10–30 ng/mL, only 2.8% of patients had distant metastases. However, for patients with ps-Tg levels within the range of 30.1–50 ng/mL this changed to 9%, while 16.9% of patients with a ps-Tg level ranging from 50.1–100 ng/mL had distant metastasis. In the group that included our cutoff point for identification of distant metastases (above 100 ng/mL), 41.6% of patients had either pulmonary or bone metastasis. Thus, patients with ps-Tg between 30.1 to 50 ng/dL were 6.9 times more likely to have metastasis than patients with the level between 10 to 30 ng/dL. This chance was 24.3 times higher if ps-Tg was above 100 ng/dL (Table 2). In the 104 patients with metastatic lesions, the majority were of the last two groups, 13.4% with a ps-Tg level ranging from 50.1 to 100 and 71.1% with ps-Tg greater than 100 (Figure 2). To evaluate the most sensitive and specific ps-Tg values to identify the presence of metastases, our analysis utilized the ROC curve (Figure 3). The cutoff value of ps-Tg related to the presence of pulmonary metastasis was 117.5 ng/mL (sensitivity: 70.2%; specificity: 71.7%; negative predictive value [NPV]: 94.3%), and the cutoff point related to bone metastasis was 150.5 ng/mL (sensitivity: 79.3%; specificity: 85.5%; NPV: 98.8%). When patients with pulmonary or bone metastases were analyzed, the cutoff point was 117.5 ng/mL (sensitivity: 70.2%; specificity: 83.7%; NPV: 93.7%). The area under the ROC curve for the ps-Tg level used for identifying metastasis was 0.836 (pulmonary), 0.847 (bone), and 0.845 (pulmonary or bone) (Table 4).

**Table 4:** Ps-Tg values to identify distant metastases in patients with differentiated thyroid cancer.

Metastasis	Cutoff (ps-Tg)	AUC	Sensitivity	Specificity	NPV	Accuracy	P value
Pulmonary metastasis	117.5 ng/mL	0.836	70.2%	71.7%	94.3%	81.4%	<0.001
Bone metastasis	150.5 ng/mL	0.847	79.3%	85.0%	98.8%	80.9%	<0.001
Pulmonary or bone metastasis	117.5 ng/mL	0.845	70.2%	83.7%	93.7%	82.0%	<0.001

Ps-Tg: postoperative stimulated thyroglobulin; AUC: area under the curve; NPV: negative predictive value.

**Figure 4:**
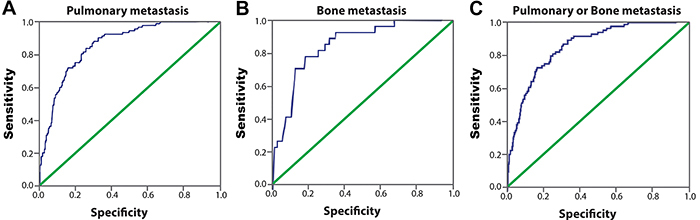
Analysis of patients over 18 years. ROC of serum postoperative stimulated thyroglobulin (ps-Tg). ROC in identifying the presence of **A**, pulmonary metastasis; **B**, bone metastasis; and **C**, either pulmonary or bone metastasis. The area under the curve (AUC) was 0.851 (pulmonary), 0.848 (bone), and 0.859 (pulmonary or bone).

In our study, 21 patients under 18 years of age were included: 15 females and 6 males. Some studies (28,29) show that this population may have a higher level of ps-Tg. However, excluding these patients, in a new analysis with 634 patients, all over 18 years old, it was possible to maintain the same cutoff without a negative impact, with increased sensitivity and specificity values (Figure 4, Table 5).

**Table 5:** Ps-Tg values to identify distant metastases in patients with differentiated thyroid cancer over 18 years.

Metastasis	Cutoff (ps-Tg)	AUC	Sensitivity	Specificity	P value
Pulmonary metastasis	117.5 ng/mL	0.851	72.1%	82.8%	<0.001
Bone metastasis	150.5 ng/mL	0.848	78.6%	81.0%	<0.001
Pulmonary or bone metastasis	117.5 ng/mL	0.859	71.9%	83.8%	<0.001

Ps-Tg: postoperative stimulated thyroglobulin; AUC: area under the curve.

**Figure 5:**
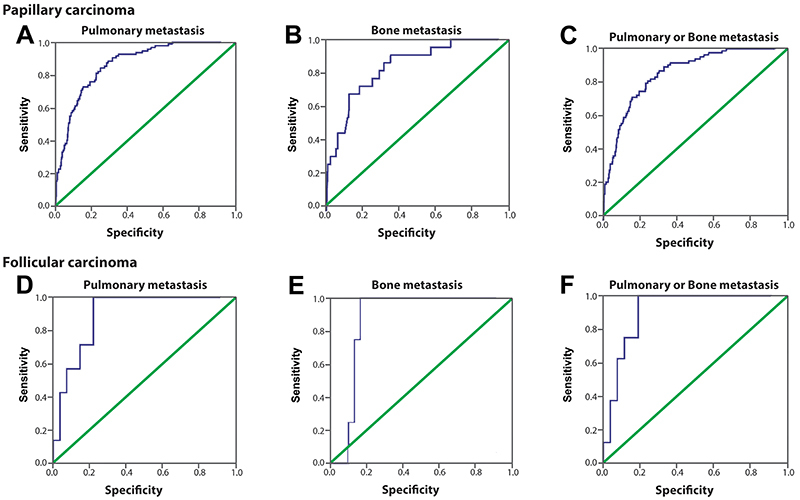
ROC of serum postoperative stimulated thyroglobulin (ps-Tg). **A**-**C**, Papillary carcinoma: ROC in identifying the presence of **A**, pulmonary metastasis; **B**, bone metastasis; and **C**, either pulmonary or bone metastasis. The area under the curve (AUC) was 0.841 (pulmonary), 0.834 (bone), and 0.849 (pulmonary and bone). **D**-**F**, Follicular carcinoma: ROC in identifying the presence of **D**, pulmonary metastasis; **E**, bone metastasis; and **F**, either pulmonary or bone metastasis. The AUC was 0.886 (pulmonary), 0.857 (bone), and 0.901 (pulmonary or bone).

Since the pattern of metastasis of papillary and follicular thyroid cancer are different, we also performed an analysis with these subgroups. Considering just patients over 18 years of age, when we analyzed these histologic subtypes separately, in the group of papillary thyroid cancer patients, the cutoff values of ps-Tg remained the same, with sensitivity values above 70% and specificity above 80% (Table 6). In the group of patients with follicular subtype, the cutoff was 138 ng/mL for metastases at both sites alone, as well as for those with bone or pulmonary metastasis (Table 6). Even if separating the groups, the area under the curve was significant, showing the discriminatory power of ps-Tg to identify distant metastases in these two types of thyroid cancer (Figure 5). We observed that in patients with the follicular pattern, the cutoff value to identify bone metastasis was lower (138 ng/mL) than the value found in papillary cancer, with sensitivity of 100% and specificity of 82.1%.

**Table 6:** Ps-Tg values to identify distant metastasis in patients with papillary and follicular thyroid cancer.

	Cutoff (ps-Tg)	AUC	Sensitivity	Specificity	P value
Papillary carcinoma					
Pulmonary metastasis	117.5 ng/mL	0.841	72.1%	82.8%	<0.001
Bone metastasis	150.5 ng/mL	0.834	72.7%	80.8%	<0.001
Pulmonary or /bone metastasis	117.5 ng/mL	0.849	71.1%	82.7%	<0.001
Follicular carcinoma					
Pulmonary metastasis	138 ng/mL	0.886	71.4%	84%	0.002
Bone metastasis	138 ng/mL	0.857	100%	82.1%	0.023
Pulmonary or /bone metastasis	138 ng/mL	0.901	75%	87.5%	0.001

Ps-Tg: postoperative stimulated thyroglobulin; AUC: area under the curve.

## Discussion

Accurate postoperative risk assessment is an important consideration for guiding initial disease management and follow-up strategies in patients with DTC. This postoperative assessment can be evaluated by a number of diagnostic strategies including measurements of serum thyroglobulin as well as neck ultrasonography and whole-body scan (4).

Postoperative serum thyroglobulin levels can be a predictor of persistent disease after surgery, according to the ATA's guidelines, but the optimal cutoff value to guide decisions in terms of appropriate radioiodine dose has not yet been well established. Several studies (11–15,22–26,30–37) have demonstrated the clinical use of the serum thyroglobulin measurement (either TSH stimulated or non-stimulated) as a tool to aid in initial risk stratification to guide treatment and predict prognosis.

Piccardo et al. (11) and Lin et al. (25) demonstrated that ps-Tg values less than 1-2 ng/mL are strong predictors of remission. Furthermore, a meta-analysis demonstrated a high negative predictive value for ps-Tg levels less than 10 ng/mL for predicting a subsequent disease-free status (26).

Conversely, some studies associate greater ps-Tg values with worse prognosis in DTC patients. In one prospective study, a ps-Tg threshold of >5 ng/mL was considered appropriate to indicate RIT (31 [Bibr B32]). Two other studies (34,35) reported that elevated Tg levels (>5-6 ng/mL) may be a predictor of failing ablation, after administration of RIT doses of 30 mCi (34) and 100 mCi (35), suggesting that higher Tg levels could be correlated with a more aggressive tumor profile. Furthermore, some studies have found an association between high ps-Tg values (>10-30 ng/mL) and poor overall survival, lower disease-free survival (11,13,33), and in some multivariate analyses, a greater risk of persistent or recurrent disease (11,12,36). Following the same rationale, a retrospective cohort study (35) showed patients with thyroglobulin levels of 27.5 μg/L or higher had a significantly increased relative risk of disease recurrence of 4.50. Piccardo et al. (11), when evaluating the ps-Tg value as a predictor of recurrent disease in patients classified as high-risk by the European Consensus (37), concluded that ps-Tg was the most important independent prognostic factor for disease persistence compared to other variables. They also suggested the introduction of a cutoff value of 50 ng/mL to promptly identify patients with risk of recurrence.

In a retrospective study, Yang et al. (19) revealed the clinical utility of the ps-Tg value as a prognostic indicator and often the only evidence available in the postoperative evaluation to indicate appropriate dose of RIT. Zhao et al. (21) emphasized the concept of diagnosing the presence of distant metastatic lesions through dynamic monitoring of changes in ps-Tg. They showed the potential of serial ps-Tg measurements in identifying these lesions much earlier, prior to RIT administration, and also as an alternative indicator to imaging modalities, especially for those with negative imaging results.

Additionally, some studies (20,23,24) found a significant difference in ps-Tg concentration in patients with or without distant metastasis. In a retrospective study (20), the ROC analysis of ps-Tg and thyroglobulin/thyroid stimulating hormone ratio showed good accuracy as diagnostic markers. They were considered useful indexes for probable metastases, especially for those with micrometastases that cannot be detected by other imaging modalities before ^131^I therapy.

Several other studies have shown that the likelihood of metastasis is higher when ps-Tg values rise above 5-10 ng/mL (16–18). However, in all these studies when the likelihood of recurrence was evaluated, patients with distant metastases were pooled with patients experiencing locoregional metastases; this may explain the lower ps-Tg levels found in these studies.

In the present study, our results suggested that a higher level of ps-Tg more accurately predicted a high risk of recurrence. We identified a cutoff point of 117.5 ng/mL with a high negative predictive value (93.7%), which reliably excluded the presence of distant metastasis in patients with DTC. This value of ps-Tg that correlated with distant metastases without including patients with cervical metastasis alone may be important to improve recurrence risk stratification and to dictate the most appropriate therapy, such as deciding on the appropriate radioiodine dose.

During postoperative evaluations, we routinely requested cervical ultrasonography. A specific ps-Tg cutoff value that correlates with distant metastasis may be useful to identify the need to request additional imaging tests prior to RIT. In addition, ps-Tg levels may be the only available evidence for the identification of occult distant metastases, which may not have been visible in imaging exams. This can help ensure against undertreating patients.

Using ROC analyses, similar to our study, Yang et al. (19) found that a ps-Tg cutoff value of 47.1 ng/mL was a good predictive index for identifying distant metastases. They also showed that if this cutoff point was measured before RIT, it could prevent under treatment of 10.26% of patients with M1 stage disease who had no evidence of distant metastasis in the pre-ablative assessment. Therefore, reassessment of ps-Tg levels could be the only available evidence to indicate a need for a higher postoperative radioiodine dose. Similar data have been reported by Li et al. (22), who suggested a cutoff point of 52.75 ng/mL (sensitivity of 78.90% and specificity of 91.70%) for predicting distant metastases during the postoperative period. Interestingly, the ps-Tg value of 117.5 ng/mL identified in our study was higher than those reported in earlier studies (19,22). This higher value may more accurately identify high-risk patients requiring more aggressive therapy. Possible explanations for this variability are: different levels of TSH at the time of ps-Tg testing, using a single measurement, which may be affected by various factors such as remnant thyroid tissue, TSH levels, the different times between thyroidectomy and the measurement of thyroglobulin, and others. In addition, care should be taken when comparing the ps-Tg cutoff between different centers. With this higher cutoff point, we obtained a high negative predictive value, with a low number of false negative results, which may have the benefit of excluding the presence of distant metastases, with an important impact on therapeutic planning and follow-up strategies.

Similar to our cutoff point, Liu et al. (23) proposed a ps-Tg value of 156 ng/mL as a predictor of distant metastasis in a pediatric population with DTC. It is still necessary to explore this difference in ps-Tg values between adult and pediatric thyroid cancer patients. Additionally, some studies comparing ps-Tg values in patients with pulmonary or bone metastasis to patients without distant metastasis found higher median values, similar to our study (20,). In a retrospective analysis, Lin et al. (20) found a significant difference in ps-Tg levels in patients with or without distant metastasis (440.6 *vs* 5.3 ng/mL). Makarewicz et al. (30) divided patients into groups, without evidence of metastasis and with local and distant metastases. A statistically significant difference was found in relation to the ps-Tg value in both groups (4.3 *vs* 97.4 ng/mL). Thyroglobulin was higher in the group of patients with distant metastases than in the group with metastases limited to cervical lymph nodes (382.9 *vs* 18.2 ng/mL).

The limitations of this study were the retrospective nature of the data used and the lack of information regarding patient follow-up. The data used were obtained from a database of a nuclear medicine service where, in the vast majority of cases, patients are referred just to perform treatment and/or exams and do not return. Other limitations were that we did not include patients with distant metastasis to atypical sites because of their small number and that we used the post-therapy whole body scan to exclude lymph node disease. We know that cervical ultrasound would be a more sensitive image to exclude lymph node metastases but unfortunately, not all patients had received this exam.

Caution should be exercised when comparing ps-Tg cutoff levels among different centers. In addition, the volume of remnant thyroid post-operatively may affect the measurements of ps-Tg. Therefore, the clinical utility of ps-Tg in the identification of distant metastasis should be validated in further studies. We should be aware that undifferentiated tumors may present low levels of thyroglobulin, which is only one of the criteria to classify patient with a high risk of recurrence.

In summary, our results confirmed the clinical value of ps-Tg for identifying distant metastases. We suggest a cutoff of 117.5 ng/mL, with high negative predictive value (93.7%), to reliably exclude the presence of both pulmonary and bone metastasis in patients with DTC. Our findings demonstrated that assessment of ps-Tg levels may be a good prognostic tool and useful for distinguishing patients who may need more aggressive therapy. Congruently determining ps-Tg levels can minimize treatment-related morbidity and unnecessary procedures. We believe that ps-Tg could be considered as a marker with good accuracy in postoperative risk stratification of DTC patients, with a possible significant impact on their management and follow-up decisions.

## Supplementary Material

Click here to view [pdf].
